# Using Implementation Science Strategy Mapping on the Age-Friendly Health System's 4Ms Journey

**DOI:** 10.1093/geroni/igab046.1409

**Published:** 2021-12-17

**Authors:** Mary Dolansky, Anne pohnert, Sherry Greenberg

**Affiliations:** 1 Case Western Reserve University, Cleveland, Ohio, United States; 2 CVS Minute Clinic, Woonsocket,, Rhode Island, United States; 3 Seton Hall University College of Nursing, Seton Hall University College of Nursing, New Jersey, United States

## Abstract

Background: Implementation science is the study of methods to promote the adoption and integration of evidence-based practices into routine health care to improve the quality of care. The purpose of this study was to use Implementation Mapping to guide the implementation of The John A. Hartford evidence-based Age-Friendly Health Systems (AFHS) 4Ms Framework: What Matters, Medications, Mentation, and Mobility. Methods: Implementation Mapping, a systematic process for planning implementation strategies, guided the 9-month integration of the 4Ms Framework in the 1,100 MinuteClinics across the US. Implementation Mapping includes five tasks: (1) conduct an implementation needs assessment and identify program adopters and implementers; (2) state adoption and implementation outcomes and performance objectives, identify determinants, and create matrices of change objectives; (3) choose theoretical methods (mechanisms of change) and select or design implementation strategies; (4) produce implementation protocols and materials; and (5) evaluate implementation outcomes. Results: The implementation plan, developed by the implementation mapping method, was carried out over 9-months. Seven implementation strategies were identified from the Expert Recommendations for Implementing Change (ERIC) project including the provision of education, electronic health record integration, internal champion facilitation, cues to action, and a dashboard to monitor progress. To date, the implementation mapping has resulted in the adoption of the 4Ms by 1145 providers (37%). Monitoring of the adoption of the 4Ms Framework and consideration of future implementation strategies is ongoing. Conclusions: Implementation Mapping provided a systematic process to develop strategies to improve the adoption, implementation, sustainment, and scale-up of the evidence-based 4Ms Framework.

